# Multiscale Structural Evolution and Digestion Kinetics of Starch in Commercial Swine Compound Feed as Affected by Steam Conditioning Temperature and Retention Time

**DOI:** 10.3390/ani16091399

**Published:** 2026-05-03

**Authors:** Junhua Wu, Fanglei Zou, Wei Wang, Liangju Wang, Hongying Wang

**Affiliations:** College of Engineering, China Agricultural University, Beijing 100083, China; junhuaw@cau.edu.cn (J.W.); flzou@cau.edu.cn (F.Z.); bs20223070638@cau.edu.cn (W.W.); wangl@cau.edu.cn (L.W.)

**Keywords:** steam conditioning, pelleted feed, starch structural reorganization, in vitro digestion kinetics, matrix constraint

## Abstract

Steam conditioning is a key hydrothermal process for ensuring the quality of pelleted commercial swine compound feed. However, the molecular evolution of starch during processing remains poorly understood, particularly under the industrial constraints of limited moisture and complex substrates. This study quantified the effects of conditioning temperature (60–90 °C) and retention time (2–4 min) on starch structural reorganization across multiple length scales and in vitro digestion kinetics in pig diets. Structural evolution exhibited a nonlinear, temperature-driven pattern, with the temperature region around 80 °C representing a critical transition in structural reorganization. Although high-temperature conditioning (90 °C) increased the initial digestion rate, a nonlinear structure–digestion response was observed: final digestibility decreased, and resistant starch content rebounded. Microstructural analyses revealed that severe heat treatment was associated with the possible formation of amylose–lipid complexes (V-type crystallinity) and a more continuous protein-associated matrix, which may have imposed additional physical constraints on enzymatic hydrolysis. Consequently, maximizing starch gelatinization does not necessarily guarantee optimal in vitro digestibility. These findings integrate multiscale structural analysis with digestion kinetics, providing a mechanistic basis for balancing energy consumption, pellet quality, and nutritional value in the feed industry.

## Simple Summary

Pelleted compound feed is widely used in modern pig production because it reduces feed waste and improves animal performance. Steam conditioning is a critical pre-pelleting step involving the application of heat and moisture to the feed mixture. However, the molecular changes occurring within feed ingredients during conditioning remain poorly understood, particularly under typical industrial conditions characterized by limited moisture and complex ingredient mixtures. In this study, a commercial swine compound feed was used to investigate how conditioning temperature and retention time affect starch structure and digestibility. By combining multiscale structural analysis with an in vitro digestion model, we found that starch structural changes were primarily driven by temperature rather than time. A critical transition occurred around 80 °C, above which starch structures underwent significant disruption. At severe processing temperatures (90 °C), starch digestion was initially faster, but the final digestibility decreased. This reduction was mainly due to the formation of dense protein-associated networks and crystalline complexes that acted as physical barriers to enzyme access. These results indicate that maximizing starch gelatinization does not necessarily translate to improved nutrient availability. Ultimately, these findings provide new insights into how feed processing conditions influence nutrient utilization, offering practical guidance for the feed industry to optimize conditioning parameters and strike an optimal balance among pellet quality, energy consumption, and nutritional value.

## 1. Introduction

Global production of compound feed reached approximately 1.396 billion tonnes in 2024, with poultry and pig feed accounting for 42.7% and 26.4% of the total volume, respectively [1]. In modern intensive farming, pelleted compound feed has become the predominant dietary form for monogastric animals due to its ability to reduce wastage, improve palatability, and enhance growth performance [2,3]. The production of pelleted compound feed involves multiple unit operations such as grinding, mixing, conditioning, and pelleting [4]. Achieving the dual optimization of physical quality and nutritional efficacy in pelleted feed depends on elucidating the three-dimensional molecular architecture and functional characteristics of macromolecules (e.g., starch) in both raw materials and final products. Crucially, this requires an understanding of how these properties evolve under diverse processing conditions (e.g., grinding, conditioning, pelleting, and extrusion) [5,6,7]. Steam conditioning, as the main hydrothermal treatment step, is a pivotal step for ensuring the physical quality (e.g., pellet durability and hardness) and nutritional efficacy of the feed [8,9]. However, in actual industrial production, the determination of conditioning parameters (temperature and time) is frequently based on practical experience, with limited mechanistic understanding of the molecular events occurring within the material during processing [10]. Specifically, there remains limited quantitative insight into how distinct process parameters drive the structural reorganization of macromolecules, such as starch and protein, within the composite matrix, thereby reshaping their physicochemical properties and nutritional value. This lack of mechanistic understanding significantly hinders the consistent and precise production of compound feed [11].

In grain-based monogastric animal diets, starch accounts for 35–60% of dry matter and serves as the main energy source [12]. It is therefore important to clarify starch gelatinization and its driving mechanisms during conditioning. However, there are fundamental differences between feed conditioning and pure starch systems or cooking environments with sufficient water. Specifically, the conditioning process is characterized by dual constraints: limited moisture and a complex matrix. Conditioning typically operates under restricted-moisture conditions (10–13% initially), with steam condensation providing an unevenly distributed moisture increment of only 4–5% [13]. As pointed out by Fukuoka et al. [14], complete starch gelatinization can only be achieved when the water-to-starch ratio exceeds 3:1. Under the restricted-moisture environment of feed conditioning, starch often undergoes only partial gelatinization or surface modification [15,16]. In addition, non-starch components in compound feed (proteins, lipids, and fiber) interact with starch in a complex manner. Previous studies have demonstrated that steam treatment induces the following effects: (i) the loosening of the starch lamellar structure and partial gelatinization; (ii) protein denaturation and the formation of a hydrophobic coating layer on granule surfaces; and (iii) the formation of V-type complexes between amylose and endogenous lipids. The resulting “physical barrier” formed by non-starch components restricts water penetration and granule swelling, thereby reshaping the starch gelatinization curve and retrogradation behavior [17,18]. It is important to note that much of the current research still relies solely on the macroscopic metric of “gelatinization degree” as an evaluation indicator [19]. Decades ago, Hale [20] observed that the relationship between the degree of starch gelatinization and animal utilization is not strictly linear. Relying solely on this single indicator makes it challenging to comprehensively elucidate the microstructural reorganization of multicomponent matrices under limited-moisture conditions and their nutritional significance.

Compound feeds subjected to hydrothermal processing, such as steam conditioning and pelleting, undergo significant microstructural alterations in the starch fraction within the feed matrix, which subsequently affect enzymatic hydrolysis kinetics in the gastrointestinal tract and overall growth performance in pigs [21,22]. However, because steam conditioning is a complex and still incompletely understood operation involving intense heat and moisture transfer, it is crucial to decipher the underlying mechanisms by which processing parameters affect nutrient availability at the microstructural level before conducting time-consuming and costly in vivo animal trials. Drawing on interdisciplinary approaches from food and feed sciences, in vitro digestion models have proven to be an essential analytical tool for evaluating starch bioavailability and screening optimal processing parameters. As demonstrated by Telles et al. [23], in vitro hydrolysis rates may help predict in vivo feed conversion efficiency in swine. This technique not only simulates the digestive environment in a relatively high-throughput manner but also precisely quantifies three key starch fractions. Rapidly digestible starch (RDS) provides a rapid energy supply, while slowly digestible starch (SDS) facilitates sustained energy delivery to the distal small intestine [24]. Furthermore, resistant starch (RS) may promote gut health through the production of short-chain fatty acids during hindgut fermentation in pigs [25]. Integrating multiscale structural characterization with in vitro digestion kinetics provides an important framework for understanding the intrinsic relationships among processing, structural evolution, and nutritional function.

Most existing studies have focused on single-starch models and have not systematically characterized the structural changes in commercial compound feed under practical conditioning conditions. To fill this gap, this study simulated the limited-moisture scenario typical of commercial swine feed production, focusing on the effects of temperature and time as two key process parameters. By employing multiscale analytical techniques—such as differential scanning calorimetry (DSC), confocal laser scanning microscopy (CLSM), X-ray diffraction (XRD), and Fourier transform infrared spectroscopy (FTIR)—we systematically characterized the structural evolution of starch, spanning from its short-range molecular order and crystalline structure to its overall microstructural morphology. Furthermore, by integrating an in vitro digestion model, we established the structure-function relationship between structural reorganization and digestion kinetics. This research not only elucidates the complex matrix constraint effects and the temperature–time thresholds for starch gelatinization but also provides critical theoretical support at the micro-mechanistic level for future in vivo trials, ultimately aiming to synergistically improve both the physical pellet quality and the nutritional value of swine feed.

## 2. Materials and Methods

### 2.1. Materials and Chemicals

The commercial swine compound feed (after blending, prior to steam conditioning) was obtained from Wellhope Foods Co., Ltd. (Shenyang, China). The ingredient composition (g/kg, as-fed basis) was as follows: corn, 510; soybean meal, 110; wheat bran, 150; wheat flour, 80; rice bran, 50; corn distillers dried grains with solubles, 50; and premix, 50.

The chemical composition of the samples was analyzed according to International Organization for Standardization (ISO) procedures: moisture (ISO 6496:1999), crude protein (ISO 5983:2009), neutral detergent fiber (NDF; ISO 16472:2006), acid detergent fiber (ADF; ISO 13906:2008), and crude ash (ISO 5984:2022). Starch content was determined using a total starch assay kit based on the glucose oxidase–peroxidase (GOPOD) method. The analyzed nutrient profile (g/kg, as-fed basis) was: moisture, 120; starch, 445; crude protein, 146.8; NDF, 156; ADF, 53; and crude ash, 50.8.

Prior to analysis and processing, all samples were ground using a high-speed universal grinder to pass through a 425 µm sieve and stored in a desiccator. The moisture content of the ground samples was determined to be 12.03 ± 0.05%.

The digestive enzymes used in the in vitro digestion included porcine pepsin (≥250 U/mg, P7000), pancreatin (8 × USP, P7545), and amyloglucosidase (≥260 U/mL, A7095), all purchased from Sigma-Aldrich (Shanghai, China). The glucose assay kit (glucose oxidase–peroxidase, GOPOD method) was obtained from Nanjing Jiancheng Bioengineering Institute (Nanjing, China). All other chemicals were of analytical grade and purchased from Sinopharm Chemical Reagent Co., Ltd. (Shanghai, China).

### 2.2. Laboratory-Scale Steam Conditioning

The experiment was conducted using a 4 × 2 full factorial design, with conditioning temperature (60, 70, 80, and 90 °C) and retention time (2 and 4 min) as the two main independent factors. Each treatment, as well as the unconditioned control, was performed in triplicate using separate batches of mash feed, serving as independent batch replicates (*n* = 3 per group).

**Figure 1:**
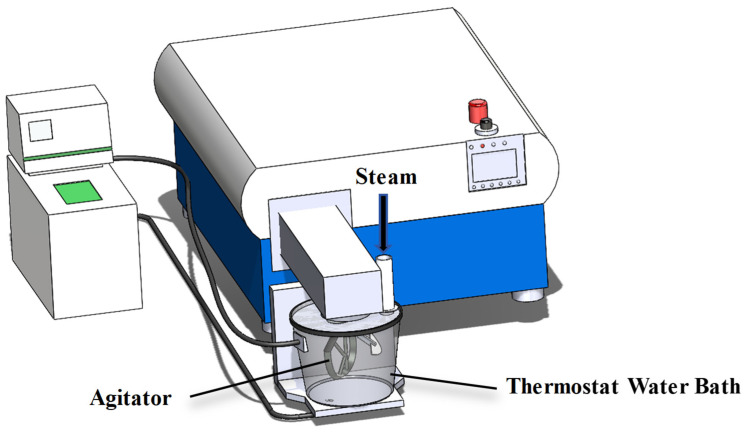
Laboratory steam conditioning system.

A laboratory-scale steam conditioning system was established, consisting of a planetary mixer (Brabender P600, Duisburg, Germany) coupled with a saturated steam generator (rated evaporation 8 kg/h, outlet pressure 0.35 MPa), as illustrated in Figure 1. To simulate industrial conditions, target temperatures were achieved by controlling the steam injection volume and the temperature of the jacketed water bath. Preliminary trials determined that steam valve opening durations of 5, 15, 25, and 35 s were required to reach 60, 70, 80, and 90 °C, respectively.

For each run, 500 g of mash with a uniform initial moisture content was loaded into the mixer chamber and continuously agitated at 90 rpm throughout the process. Saturated steam was injected for the predetermined duration. Timing commenced immediately upon the initiation of steam injection, and the retention time (defined as the steam injection duration plus the holding phase) was maintained at 2 or 4 min. During the retention phase, the target temperature was consistently maintained by the preset jacketed water bath. In this industrially simulated process, the final moisture content of the conditioned mash was thermodynamically coupled with the target temperature, as it was strictly determined by the condensation of the injected steam. Despite this, the moisture remained within the typical industrial “limited moisture” range (approximately 15–18%), well below levels required for complete starch gelatinization. The control group consisted of the same initial mash feed, which was not subjected to any water bath heating, steam injection, or mechanical agitation, but was instead sealed and kept at room temperature.

Upon completion, both the conditioned samples and the unconditioned control were discharged, spread in a thin layer, and subjected to natural air cooling and drying for 12 h at 25 ± 2 °C and 45–55% RH to return to a moisture content of approximately 12%. This step closely mimics the actual industrial cooling process, where the free water added during steam conditioning naturally evaporates. The dried samples were then ground using a cyclone sample mill with a 212 µm sieve, sealed in polyethylene bags, and uniformly stored at 4 °C until further analyses.

### 2.3. Microstructural Characterization (SEM and CLSM)

Surface morphology was characterized using a scanning electron microscope (SU3500, Hitachi, Tokyo, Japan) at an accelerating voltage of 10 kV. The dried powders were mounted onto aluminum stubs using double-sided conductive tape and sputter-coated with a thin layer of gold to improve conductivity and image quality. Micrographs were acquired at 1000× magnification.

The microstructure and spatial distribution of starch and protein were characterized using a confocal laser scanning microscope (LSM 800, Carl Zeiss, Jena, Germany). The staining procedure was performed according to the methods described by Ma et al. [26] and Gu et al. [27]. Briefly, approximately 1.0 g of the sample was dispersed in 5 mL of deionized water and soaked for 4 h to ensure hydration. Subsequently, 300 μL of the hydrated suspension was mixed with 150 μL of fluorescent dyes (consisting of 75 μL of fluorescein isothiocyanate (FITC) at 0.2 mg/mL and 75 μL of Rhodamine B at 0.02 mg/mL), yielding a sample-to-dye volume ratio of 2:1. The mixture was incubated in the dark for 30 min. To preserve the fragile spatial distribution of the starch–protein matrix and avoid structural artifacts caused by centrifugation, no washing step was performed. Finally, 30 μL of the stained suspension was pipetted onto a standard glass microscope slide and gently covered with a coverslip. No additional pressure was applied to the coverslip to minimize compression artifacts. Imaging was performed using dual excitation wavelengths: 488 nm for FITC and 561 nm for Rhodamine B. In the obtained CLSM images, starch granules were visualized as distinct green structures (stained by FITC), while the protein matrix appeared as red regions distributed around the starch granules (stained by Rhodamine B).

### 2.4. Differential Scanning Calorimetry (DSC)

Thermal transitions were profiled by differential scanning calorimetry (DSC 214; Netzsch, Selb, Germany) following Huang et al. [28] with minor adjustments. Approximately 5.0 mg (dry basis) of the milled sample was weighed into a standard hermetic aluminum pan, combined with deionized water at a 1:3 (*w*/*w*) ratio, and hermetically sealed using a sample press to prevent any moisture loss during heating. Pans were equilibrated at 25 °C for 12 h to allow complete hydration. Scans were performed from 20 to 130 °C at 10 °C/min using an empty pan as the reference. Onset (*T*_o_), peak (*T*_p_) and conclusion (*T*_c_) temperatures, together with gelatinization enthalpy (ΔH), were determined from DSC thermograms using Proteus software (version 7.0; Netzsch, Germany).

The degree of gelatinization (DG) was calculated using the following equation:

(1) $$DG(\%)=(1-\frac{\Delta H_{treated}}{\Delta H_{control}})\times 100\%$$

where $\Delta H_{treated}$ and $\Delta H_{control}$ represent the gelatinization enthalpy for the steam-conditioned and control samples, respectively.

### 2.5. X-Ray Diffraction (XRD)

The samples were analyzed using an X-ray diffractometer (XD-3, Purkinje General Instrument Co., Ltd., Beijing, China) to collect diffraction patterns. The instrument was operated with Cu-Kα radiation at 36 kV and 20 mA, scanning from 4 to 40° (2*θ*) at a rate of 2.5°/min. Diffraction profiles were processed using MDI Jade 6.5 software. Relative crystallinity (RC) was determined by the area method [28]. Specifically, the crystalline regions were defined by the characteristic diffraction peaks observed at 2*θ* values of approximately 5.6°, 15°, 17°, 18°, 20°, and 23°, while the broad underlying background halo was treated as the amorphous region. RC was calculated as the ratio of the integrated area of these crystalline peaks to the total diffraction area.

### 2.6. Fourier Transform Infrared (FTIR)

Short-range molecular order before and after steam conditioning was assessed by FTIR spectroscopy (Nicolet 6700, Thermo Fisher Scientific, Waltham, MA, USA). Experimental methods followed Ma et al. [26]. Approximately 2 mg of dry sample was thoroughly mixed and ground with 200 mg of dry potassium bromide (KBr), then pressed into a transparent pellet under a pressure of 10 MPa for 1 min. Spectral data were collected in transmission mode at a resolution of 4 cm^−1^, scanning from 4000 to 400 cm^−1^. For each sample, 32 cumulative scans were averaged to enhance the signal-to-noise ratio.

The spectral data were processed using OMNIC 8.2 software (Thermo Fisher Scientific). Specifically, the spectra in the range of 1200–800 cm^−1^ were baseline-corrected, smoothed, and deconvoluted using a half-bandwidth of 19 cm^−1^ and an enhancement factor of 1.9. Short-range ordering of starch chains was evaluated using the 1047/1022 cm^−1^ band-intensity ratio (*R*_1047/1022_), while the relative double-helix content was indicated by the 1022/995 cm^−1^ ratio (*R*_1022/995_).

### 2.7. Rapid Visco Analyzer (RVA)

The pasting properties of untreated and steam-conditioned samples were determined using a Rapid Visco Analyzer (RVA-TecMaster, Perten Instruments, Warriewood, Australia). The workflow was adapted from the methods of Hong et al. [29] with minor modifications. Since all samples were previously standardized to a uniform moisture content of approximately 12%, an aliquot of 5.0 g was weighed, combined with 25.0 mL of deionized water, and transferred into an aluminum sample cylinder. The test program was set as follows: equilibration at 50 °C for 1 min, heating to 95 °C at 12 °C/min, holding at 95 °C for 2.5 min, cooling to 50 °C at the same rate, and finally holding at 50 °C for 2 min. The paddle speed was maintained at 160 rpm throughout the measurement, following an initial speed of 960 rpm for the first 10 s. The instrument automatically recorded the viscosity curve as a function of temperature and time using Thermocline for Windows (TCW3) software (version 3.15, Perten Instruments). The software calculated a series of gelatinization parameters based on this curve, including pasting temperature, peak viscosity, trough viscosity, breakdown, final viscosity, and setback.

### 2.8. In Vitro Starch Digestibility and Hydrolysis Kinetics

The in vitro starch digestibility was determined based on the method of Englyst et al. [30], with modifications described by Martens et al. [31]. Briefly, 1.0 g of the sample was suspended in 10 mL of 0.05 M HCl solution containing pepsin (5 mg/mL) to achieve an acidic environment (approx. pH 3.0). The mixture was incubated at 37 °C in a shaking water bath (160 rpm) for 30 min to simulate gastric hydrolysis. Subsequently, the pH of the digesta was adjusted to approximately 6.0 by adding 5.0 mL of sodium acetate buffer (0.5 M) to simulate intestinal conditions, followed by the addition of 5.0 mL of a mixed enzyme solution. The mixed enzyme solution was prepared by dispersing 3.0 g of pancreatin in 20 mL of deionized water, centrifuging to collect 15 mL of the supernatant, and uniformly mixing it with 0.7 mL of amyloglucosidase. The mixture was then incubated at 37 °C with continuous shaking for 300 min.

Aliquots (0.1 mL) were withdrawn at 0, 10, 20, 30, 60, 90, 120, 180, 240, and 300 min, immediately quenched with 0.9 mL of absolute ethanol, and then centrifuged (5000 rpm, 10 min, 25 °C) to obtain supernatants. Glucose was quantified using a commercial GOPOD kit (Nanjing Jiancheng Bioengineering Institute, Nanjing, China) via a single-point calibration method. The absorbance of the digests, alongside a reagent blank and a reference standard (5.55 mmol/L D-glucose), was measured at 505 nm using a microplate reader. Based on cumulative glucose release, RDS was defined as starch hydrolyzed within 0–20 min, SDS as starch hydrolyzed from 20 to 120 min, and RS as the fraction remaining after 120 min. The calculation formulae were as follows:

(2) $$RDS(\%)=(G_{20}-G_{0})\times 0.9\times 100/TS$$

(3) $$SDS(\%)=(G_{120}-G_{20})\times 0.9\times 100/TS$$

(4) RS(%)=(1−RDS−SDS)×100

where G_0_, G_20_, and G_120_ are the glucose released at 0, 20, and 120 min; TS is the total starch content; and 0.90 is the factor converting glucose to starch equivalents.

(5) $$C_{t}=C_{\infty }(1-e^{-k\cdot t})$$

where *C*_t_ (%) is the fraction of starch hydrolyzed at time t (min), *C*_∞_ (%) is the asymptotic (end-point) digestible fraction, and *k* is the first-order digestion rate constant.

### 2.9. Statistical Analysis

Data (mean ± SD, *n* ≥ 3) were analyzed using SPSS 25.0. After confirming the statistical assumptions of normality (Shapiro–Wilk test) and variance homogeneity (Levene’s test), a one-way ANOVA followed by Duncan’s multiple range test was applied to evaluate significant differences (*p* < 0.05) among all groups, including the untreated control. Subsequently, a two-way ANOVA was performed exclusively on the steam-conditioned samples to determine the main effects of temperature and time, as well as their interactive effects.

## 3. Results

### 3.1. Microstructural Characterization (SEM and CLSM)

**Figure 2:**
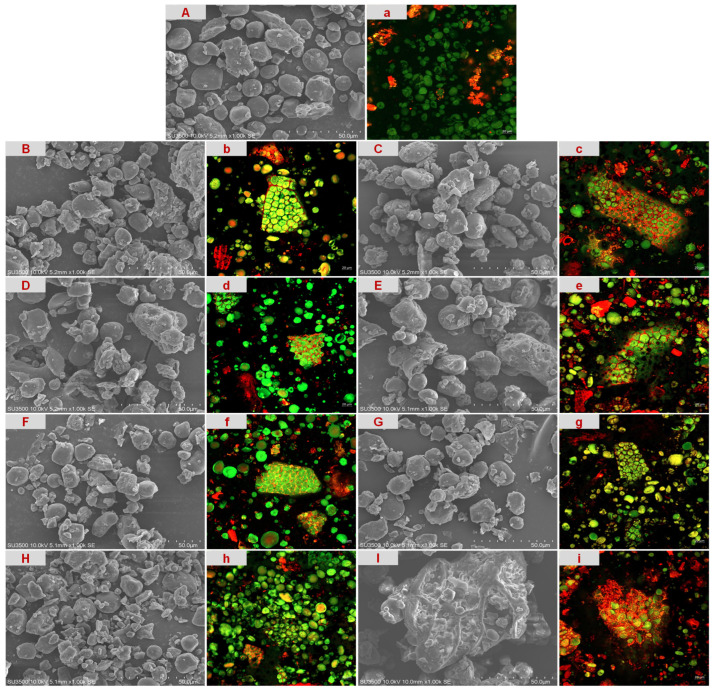
Microstructural characterization of the compound feed samples. (**A**–**I**) Scanning electron microscopy (SEM) images; (**a**–**i**) confocal laser scanning microscopy (CLSM) images (starch granules in green fluorescence, proteins in red fluorescence). Samples are control (**A**,**a**), 60 °C/2 min (**B**,**b**), 60 °C/4 min (**C**,**c**), 70 °C/2 min (**D**,**d**), 70 °C/4 min (**E**,**e**), 80 °C/2 min (**F**,**f**), 80 °C/4 min (**G**,**g**), 90 °C/2 min (**H**,**h**), and 90 °C/4 min (**I**,**i**).

The microstructural characteristics of the feed samples were evaluated using SEM and CLSM. The SEM micrographs (Figure 2A–I) revealed three typical starch morphologies in the unconditioned control sample (Figure 2A): irregular polyhedral maize starch with sharp edges, lenticular wheat A-granules, and near-spherical wheat B-granules [26,32]. The overall surface of the sample appeared smooth with well-defined boundaries, showing no signs of rupture or collapse. Trace amounts of flocculent material were observed adhering between the granules, which were presumed to be a protein matrix. Following steam conditioning at 60 °C and 70 °C (Figure 2B–E), slight morphological changes occurred at the edges of some granules, manifesting as localized roughness and slight undulations, while the integrity of the granules was maintained. At 80 °C and 90 °C (Figure 2F–H), surface shrinkage of the granules became more significant, and minor collapse was observed locally. The contact between granules increased, resulting in small-scale agglomeration. When the treatment conditions reached 90 °C for 4 min (Figure 2I), the continuous phase between granules increased significantly, with irregular network structures forming locally and some intact granules becoming embedded. With increasing conditioning temperature and time, the surface of the granules tended to become rough and undulating, and the adhesion and aggregation effects became more pronounced.

CLSM imaging (Figure 2a–i) further revealed the changes in component distribution. In the unconditioned control (Figure 2a), the starch granules (green) had clear contours and were mainly dispersed individually. The protein signal (red) was weak and scattered in a dot-like pattern on the surface or in the interstitial spaces. Following conditioning (Figure 2b–h), starch granules appeared clustered in some areas, and the surface of the granules transitioned from smooth to slightly undulating. The protein signal gradually changed from a punctate structure to a thin layer or network, forming a coating-like layer along the periphery of the granules and creating thin bridges between them. At the same time, localized porous-like regions enriched with surface protein signals were observed. At 90 °C for 4 min (Figure 2i), protein signals formed a relatively continuous coating layer on the granule surface, extending into a fine network structure across granules, and the boundaries between granules tended to become blurred. With increasing processing intensity, the particle morphology and component distribution transitioned from a dispersed state to a connected and networked state. The SEM and CLSM observations showed good consistency; together, these observations suggest that increasing processing intensity promoted a progressive reorganization of the protein phase from a discontinuous punctate distribution to a more continuous coating- and bridge-like matrix associated with the starch granules, especially under the 90 °C/4 min treatment.

### 3.2. Thermal Properties and Degree of Gelatinization

As shown in Table 1, steam conditioning significantly altered the thermal transition properties and DG of the feed samples. According to the two-way ANOVA, conditioning temperature exhibited a highly significant main effect on all thermal parameters and DG (*p* < 0.001), whereas retention time and their interaction showed no significant effects (*p* > 0.05). The control exhibited *T*_o_, *T*_p_, and *T*_c_ of 51.17 °C, 91.47 °C, and 99.07 °C, respectively, with a Δ*H* of 2.87 J/g. At conditioning temperatures of 60 and 70 °C, no significant differences were observed in thermal parameters or DG compared to the control (*p* > 0.05).

However, when the conditioning temperature was increased to 80 and 90 °C, *T*_o_, *T*_p_, and *T*_c_ significantly decreased (*p* < 0.05), accompanied by a marked reduction in Δ*H*. Specifically, at 90 °C, the *T*_o_ of the residual endothermic transition shifted to the range of 30–34 °C, and Δ*H* decreased to 0.76–0.85 J/g, indicating that the majority of starch had gelatinized, with the DG reaching 73.62%. Contrary to previous assumptions, extending the retention time from 2 to 4 min at these higher temperatures did not result in a statistically significant further decrease in Δ*H* or increase in DG (*p* > 0.05), confirming that the thermal transition was almost exclusively driven by temperature.

### 3.3. Crystalline Structure

**Figure 3:**
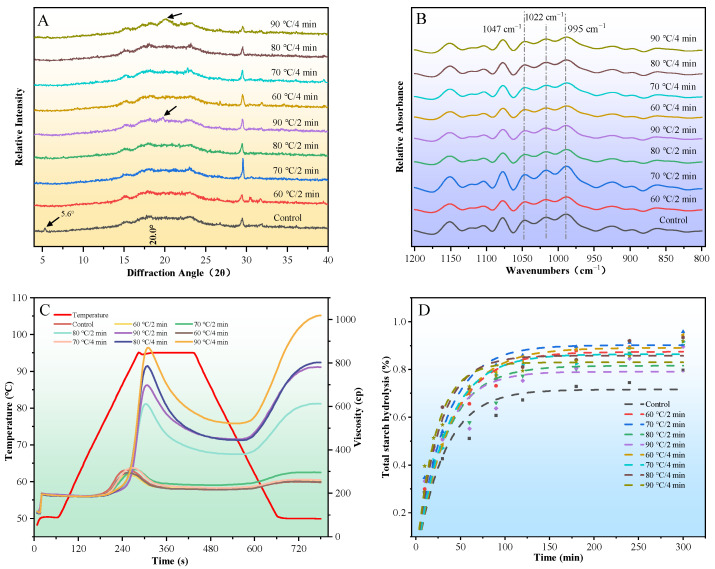
X-ray diffraction patterns (**A**), deconvoluted FTIR spectra (**B**), pasting curves (**C**), and starch hydrolysis curves (**D**) of compound feed samples.

As shown in Figure 3A, the unconditioned control exhibited a typical C-type diffraction pattern, characterized by distinct diffraction peaks at 2*θ* ≈ 5.6°, 15°, 17°, 18°, and 23°. After steam conditioning, the low-angle peak at 5.6° (characteristic of B-type polymorphs) disappeared, indicating the preferential melting of B-type crystallites. With increasing conditioning temperature, the diffraction pattern shifted towards an A-type profile dominated by peaks at 15°, 17°, 18°, and 23°. Notably, at 90 °C (2 or 4 min), a new weak peak appeared at 2*θ* ≈ 20°, indicating the formation of amylose–lipid complexes (V-type crystallinity). Regarding the RC (Table 2), the two-way ANOVA revealed no significant main effects of conditioning temperature or retention time (*p* > 0.05). Despite the severe thermal treatments, the RC values were maintained at a relatively stable level across all groups, with no statistically significant differences observed among the treatments.

### 3.4. Short-Range Ordered Structure

As shown in Figure 3B, the FTIR spectral profiles of all treated samples remained macroscopically consistent with the control, indicating no formation of new functional groups. However, subtle variations were discernible within the starch fingerprint region (1200–800 cm^−1^). To quantitatively capture these variations, the absorbance ratios *R*_1047/1022_ (reflecting the degree of short-range order) and *R*_1022/995_ (indicating the amorphous proportion) were calculated [32,33].

As presented in Table 2, two-way ANOVA revealed that conditioning temperature had a highly significant main effect on both ratios (*p* < 0.001), while retention time significantly affected only *R*_1047/1022_ (*p* < 0.001). Under the 2 min treatment, *R*_1047/1022_ decreased significantly from 1.44 (control) to 1.05 at 80 °C and 1.06 at 90 °C (*p* < 0.05). Concurrently, *R*_1022/995_ increased to a peak of 0.51 in the 80 °C/2 min group, indicating that intense, short-term steam conditioning severely disrupted the short-range molecular order and increased amorphous characteristics. Notably, significant temperature × time interaction effects were observed for both ratios (*p* < 0.05). This interaction demonstrates that extending the conditioning duration to 4 min mitigated the structural disruption at higher temperatures. For instance, the *R*_1047/1022_ value of the 80 °C/4 min group (1.33) was significantly higher than its 2 min counterpart, and the 70 °C/4 min group (1.46) remained statistically comparable to the unconditioned control (*p* > 0.05). This buffering effect suggests that prolonged moist-heat processing may facilitate a degree of structural rearrangement or annealing within the starch granules.

### 3.5. Pasting Properties

Figure 3C and Table 3 illustrate the pasting profiles and corresponding parameters of the samples. The pasting curves of the low-temperature groups (60–70 °C, 2–4 min) overlapped extensively with that of the unconditioned control, with no significant differences observed in any parameter (peak viscosity, trough viscosity, final viscosity, breakdown, setback, or pasting temperature; *p* > 0.05).

At higher conditioning temperatures (80–90 °C), all pasting parameters increased relative to the control. Specifically, the pasting temperature rose from 75.78 °C (control) to 79.07 °C (90 °C/4 min). Peak viscosity, trough viscosity, breakdown, final viscosity, and setback all exhibited substantial increases under intense treatment.

According to the two-way ANOVA, conditioning temperature had a highly significant main effect on all pasting parameters (*p* < 0.001), and significant temperature × retention time interactions were observed for all viscosity parameters (*p* < 0.05 to *p* < 0.001), indicating that the effect of treatment duration depends strongly on temperature. At a given temperature, extending the retention time from 2 to 4 min at 80 °C did not significantly alter the parameters (*p* > 0.05), whereas at 90 °C, the 4 min treatment further increased peak viscosity, final viscosity, and setback (*p* < 0.05), highlighting the combined influence of high temperature and prolonged conditioning.

### 3.6. In Vitro Starch Digestibility and Hydrolysis Kinetics

The in vitro hydrolysis curves of the native and steam-conditioned samples are presented in Figure 3D, with the corresponding kinetic parameters summarized in Table 4. All hydrolysis curves exhibited a typical biphasic pattern characterized by a rapid initial hydrolysis rate followed by a plateau after approximately 100 min. Unlike isolated cereal starches, which typically reach equilibrium within 60 min [33], the compound feed samples in this study demonstrated slower digestion kinetics, likely attributable to the barrier effect of the complex feed matrix.

The unconditioned control contained 32.31% RDS, 23.70% SDS, and 44.00% RS. According to Duncan’s multiple range test (Table 4), the RDS content numerically fluctuated among treatments but showed no statistically significant differences compared to the control (*p* > 0.05). However, the SDS and RS fractions were highly sensitive to the conditioning temperature. At mild conditioning temperatures (60 and 70 °C), the SDS content significantly increased to 35.91–40.63%, accompanied by a marked reduction in RS content to 24.59–30.37% (*p* < 0.05). This suggests a clear conversion of resistant fractions into slowly digestible fractions under moderate moist-heat conditions. Notably, at elevated temperatures (80 and 90 °C), the RDS, SDS, and RS fractions remained statistically comparable to the unconditioned control (*p* > 0.05). This distinct phenomenon indicates that structural rearrangement or hydrothermal annealing likely occurs under high thermal input, partially restoring the matrix’s resistance to enzymatic hydrolysis.

Regarding the kinetic analysis, the first-order kinetic model provided a good fit for all hydrolysis curves (*R*^2^ ≥ 0.90). Compared to the control (68.61%), the *C*_∞_ significantly increased across all steam-conditioned groups (*p* < 0.05), peaking at 89.88% in the 70 °C/2 min group. However, the *k* value reached 4.62 at 90 °C for 4 min, which was significantly higher than that of the control group (*p* < 0.05). Crucially, the two-way ANOVA performed exclusively on the treated groups revealed that conditioning temperature exerted a significant main effect on SDS, RS, *C*_∞_, and *k* (*p* < 0.05 to *p* < 0.001). Conversely, retention time and the temperature × time interaction showed no significant effects on any of the digestion fractions or kinetic parameters (*p* > 0.05). These statistical results firmly indicate that the steam conditioning-induced disruption of the starch granule structure, which alters the accessibility of α-amylase to the substrate, is overwhelmingly driven by the thermal input rather than the duration of the treatment.

## 4. Discussion

Unlike previous studies based predominantly on purified starch or single-grain models [26,28], the present work examined starch transformation during steam conditioning in a commercial swine compound feed matrix, thereby capturing starch behavior in a realistic moisture-limited and multicomponent processing environment. The results indicate that, under such restricted-moisture conditions, starch destructuring did not follow the more continuous gelatinization pattern often described for isolated starch systems [34], but instead showed a clear threshold-like response to thermal input. Across the multiscale analyses, conditioning temperature emerged as the primary driver of structural transition, whereas the effect of retention time was more limited and became evident mainly under severe treatment conditions. This pattern further suggests that starch reorganization in the compound feed matrix was not determined by thermal input alone but was also likely influenced by the surrounding non-starch matrix.

The results of the multiscale analyses collectively indicate that a clear threshold-like structural response emerged around 80 °C in this water-limited compound feed matrix. Unlike the obvious thermal transition of pure cereal starch at 60–70 °C under sufficient-water conditions [14,15], the higher apparent transformation threshold observed in this study suggests that starch structural reorganization requires a higher thermal input in low-water, multicomponent systems [35]. Under mild conditioning (60–70 °C), thermodynamic parameters and crystalline structures remained statistically stable. Morphological alterations were restricted to preliminary granular undulation and early-stage protein redistribution, indicating a state of mild perturbation rather than extensive restructuring [36]. In contrast, elevating the temperature to 80–90 °C triggered a coordinated transformation across multiple structural levels, characterized by profound reductions in Δ*H* and short-range order, alongside intensified inter-granule adhesion and degradation [37].

Interestingly, this heat treatment above the threshold did not merely result in a unidirectional weakening of the native ordered structure; instead, it was accompanied by interfacial reconstruction and molecular complexation. A notable feature of this reorganization was the apparent divergence between characterization results at different structural scales: although DSC indicated that the DG exceeded 70% at 90 °C, the RC measured by XRD remained statistically unchanged. This stability was accompanied by the emergence of a weak V-type diffraction peak (2*θ* ≈ 20°), suggesting that the loss of native A/B-type crystalline regions may have been partially compensated by the formation of new ordered structures. Unlike previous reports showing that overheated steam treatment usually leads to a significant decrease in the crystallinity of pure cereal starches [26,33], the difference observed in this study more likely reflects the coexistence of structural disruption and new structure formation under complex matrix conditions. This phenomenon suggests that amylose released during severe processing may interact with endogenous lipids to form V-type complexes, which is broadly consistent with Ye et al. [36], who reported that high temperatures promote the formation of inclusion structures between the helical cavities of amylose and endogenous lipid fragments. Meanwhile, CLSM revealed a pronounced spatial redistribution of protein-related signals, which gradually changed from dispersed puncta to a more continuous interfacial distribution, partially surrounding the structurally perturbed starch granules. This morphological evolution reflects the protein–starch interfacial behavior observed in complex food systems [36,38]; in our matrix, this reorganized protein phase may have physically limited excessive granule expansion. In addition, the significant temperature × time interaction observed in FTIR, where the loss of short-range order was partially buffered during prolonged heating at 70–80 °C, suggests that local chain rearrangement may have occurred under limited-moisture conditions. This behavior is consistent with the typical hydrothermal annealing mechanism [38]. Therefore, severe conditioning did not simply push this matrix into a completely disordered state but instead led it into a reorganized composite state characterized by the weakening of the native structure, reconstruction of local order, and concomitant interfacial reconstruction, thereby providing a structural basis for subsequent rheological behavior and digestive response.

Severe steam conditioning markedly altered the macroscopic rheological behavior of the feed matrix, as reflected by a delayed pasting onset and a substantial increase in ultimate paste viscosity. Consistent with Hu et al. [38], this distinctive “thermal delay” may reflect matrix reorganization; as suggested by our morphological observations, we hypothesize that aggregated proteins may have acted as a physical barrier, potentially restricting initial water penetration [39]. Conversely, the pronounced increase in paste viscosity accompanying severe treatment may be associated with greater starch chain mobility and leaching, together with stronger interactions between starch and denatured protein fragments during cooling, thereby promoting the formation of a more continuous and cohesive matrix. From a processing perspective, this reorganized network holds potential technological implications for improving interparticle adhesion—thereby potentially enhancing the pellet durability index and reducing fines—although these macroscopic benefits require further direct mechanical validation [40]. However, the high viscosity induced at 90 °C indicates an important “nutritional trade-off”. From a manufacturing standpoint, high viscosity indicates good pellet quality, but physiologically, it has a dual effect. Gerrits et al. [24] indicated that increased digesta viscosity significantly slows gastric emptying and inhibits enzyme contact with the substrate. In weaned piglets whose feed intake is already limited, such satiety can reduce intake and limit growth performance. However, for pregnant sows requiring energy limitation, prolonged satiety due to delayed emptying has a positive management value. Optimizing steam conditioning parameters must therefore take into account the physiological stage of the target animal, seeking an optimal balance between “pellet quality” and “intestinal kinetics”.

An in vitro digestion model was used to quantitatively evaluate how the above structural reorganization affected starch hydrolysis. The kinetic results showed a nonlinear digestive response, indicating that a faster initial hydrolysis rate did not necessarily lead to a greater final extent of hydrolysis. For example, the 90 °C group showed the highest k value, but its *C*_∞_ was lower than that of the 70 °C group, and the RS content increased again after the intermediate-temperature range. Under mild conditioning (60–70 °C), the RDS fraction remained stable, whereas SDS increased significantly and RS decreased. This mainly reflected a redistribution between the RS and SDS fractions, suggesting that part of the resistant fraction may have shifted toward slowly digestible behavior. Unlike the hydrolysis pattern commonly reported for pure starch under excess-water conditions [14,22], the mild treatments in the present study were close to, but did not fully exceed, the major gelatinization transition range identified by DSC, and therefore only partially disrupted the native ordered structure. This difference not only reflects the effect of thermal input but also highlights the role of the compound feed matrix, in which partial loosening of starch structure may have occurred while much of the granule architecture remained preserved.

In contrast, high-temperature treatment (80–90 °C) produced a nonlinear digestive response, as more extensive structural disruption did not result in a higher in vitro hydrolysis endpoint. Although the 90 °C treatment caused more pronounced disruption of the native crystalline structure and showed the highest initial k, its final in vitro digestibility was lower than that of the 70 °C group, and the RS content increased again. This digestive pattern was generally consistent with the microstructural results. At higher thermal input, the system showed a greater extent of structural disintegration; however, the weak reflection at 2*θ* ≈ 20° in XRD suggested the possible formation of amylose–lipid complexes, while CLSM showed a more continuous protein-associated coating and bridge-like matrix around and between the particles. This change in protein distribution may be relevant because it suggests that thermal processing altered not only the starch structure itself but also the organization of the surrounding matrix. We hypothesize that this more continuous protein-associated layer around starch granules may have acted as a physical barrier, potentially limiting enzyme access during the later stage of hydrolysis, which aligns with the lower final hydrolysis extent observed in the high-temperature groups. However, because protein hydrolysis kinetics and matrix permeability were not directly quantified in the present study, the restrictive effect of this protein-associated network should be regarded as a highly plausible mechanistic hypothesis rather than a directly demonstrated mechanism.

This nonlinear response may have potential implications for nutrient utilization in animals at different physiological stages [41,42]. For example, the increase in RS may be beneficial for adult sows with greater reliance on hindgut fermentation, whereas in young animals with an immature digestive tract, such as weaned piglets, the lower hydrolysis endpoint may reduce energy utilization efficiency [43]. However, these implications should be interpreted with caution because they were not directly validated in vivo. In addition, several limitations of the present in vitro model should be acknowledged. The static digestion model did not simulate the continuous removal of the protein coating by pepsin under physiological gastrointestinal conditions, did not quantitatively distinguish the relative contributions of intrinsic starch changes and matrix encapsulation effects, and did not provide direct quantitative evidence for V-type complexes. Future studies should combine starch and protein digestion kinetics with in vivo validation to better evaluate the potential processing risks associated with excessive thermal input. Overall, the present results indicate that the effect of steam conditioning on starch digestion in a compound feed system is nonlinear: higher conditioning temperature was associated with faster early hydrolysis kinetics, but the accompanying structural reorganization may partly limit the final extent of hydrolysis.

## 5. Conclusions

Steam conditioning of commercial swine compound feed showed a critical transition in structural reorganization around 80 °C, above which a nonlinear digestive response became evident. Mild conditioning (60–70 °C) partially disrupted the native ordered structure while preserving most of the granular architecture, and was associated with the highest in vitro digestion endpoint observed in the present study (*C*_∞_ = 89.88% at 70 °C), together with a redistribution from resistant starch (RS) to slowly digestible starch (SDS). In contrast, severe conditioning at 90 °C showed the highest initial hydrolysis rate constant (*k*), consistent with more extensive disruption of the native crystalline structure, yet yielded a lower final digestibility (*C*_∞_ = 82.99%) and an increase in RS content. This apparent structural–nutritional decoupling may be related to heat-induced matrix reorganization, including possible amylose–lipid complex formation (V-type crystallinity) and a more continuous denatured protein-associated network, which may have contributed to constraining the later stage of hydrolysis. Therefore, a higher degree of structural disruption or starch gelatinization does not necessarily correspond to a higher final extent of in vitro digestion, highlighting the need to balance thermal processing intensity with the intended nutritional outcome.

## Figures and Tables

**Table 1:** Effects of steam conditioning temperature and retention time on the thermal properties and degree of gelatinization of swine compound feed.

Samples	*T*_o_ (°C)	*T*_p_ (°C)	*T*_c_ (°C)	∆*H* (J/g)	DG (%)
Control	51.17 ± 4.40 a	91.47 ± 2.12 a	99.07 ± 2.70 a	2.87 ± 0.19 a	-
60 °C/2 min	49.87 ± 1.97 ab	95.25 ± 6.45 a	104.25 ± 3.55 a	2.83 ± 0.17 a	4.60 ± 3.08 c
70 °C/2 min	46.03 ± 2.95 bc	96.75 ± 3.05 a	100.00 ± 0.20 a	2.90 ± 0.06 a	1.73 ± 1.17 c
80 °C/2 min	44.03 ± 4.75 c	83.00 ± 13.68 ab	89.17 ± 14.97 ab	1.83 ± 0.13 b	36.29 ± 4.61 b
90 °C/2 min	30.63 ± 2.57 d	72.80 ± 14.20 bc	81.85 ± 12.35 bc	0.85 ± 0.36 c	70.33 ± 12.65 a
60 °C/4 min	47.85 ± 1.35 abc	88.10 ± 2.80 a	96.20 ± 3.00 ab	2.52 ± 0.21 a	12.21 ± 7.32 c
70 °C/4 min	44.90 ± 1.44 bc	88.20 ± 6.84 a	95.57 ± 6.68 ab	2.66 ± 0.42 a	13.07 ± 6.86 c
80 °C/4 min	43.63 ± 1.40 c	83.00 ± 4.87 ab	95.27 ± 5.00 ab	1.89 ± 0.06 b	33.97 ± 2.16 b
90 °C/4 min	33.57 ± 1.01 d	63.33 ± 8.20 c	72.40 ± 10.17 c	0.76 ± 0.10 c	73.62 ± 3.55 a
Two-way ANOVA (F-value) for treated groups only					
Conditioning temperature (3)	52.23 ***	10.53 ***	9.02 ***	94.25 ***	142.51 ***
Retention time (1)	0.02	3.24	1.32	2.34	3.84
Conditioning temperature × retention time (3)	1.15	0.38	1.04	0.78	1.34

*T*_o_, onset temperature; *T*_p_, peak temperature; *T*_c_, conclusion temperature; Δ*H*, gelatinization enthalpy; DG, degree of gelatinization; ANOVA, analysis of variance. Data are presented as mean ± SD (*n* = 3). Different lowercase letters within a column indicate significant differences among all groups, including the untreated control, determined by Duncan’s multiple range test (*p* < 0.05). Two-way ANOVA (*F*-value) was performed on the steam-conditioned groups only to evaluate the main effects of conditioning temperature (*df* = 3), retention time (*df* = 1), and their interaction (*df* = 3). Significance levels: *** *p* < 0.001.

**Table 2:** Effects of steam conditioning temperature and retention time on the relative crystallinity and short-range ordered structure of swine compound feed.

Samples	RC (%)	*R*_1022/995_	*R*_1047/1022_
Control	12.06 ± 3.54 a	0.42 ± 0.01 c	1.44 ± 0.01 a
60 °C/2 min	11.55 ± 1.03 a	0.40 ± 0.04 c	1.36 ± 0.00 ab
70 °C/2 min	11.79 ± 2.92 a	0.45 ± 0.05 b	1.26 ± 0.03 bc
80 °C/2 min	8.18 ± 0.62 a	0.51 ± 0.01 a	1.05 ± 0.08 d
90 °C/2 min	10.55 ± 1.05 a	0.49 ± 0.03 a	1.06 ± 0.13 d
60 °C/4 min	11.25 ± 3.98 a	0.44 ± 0.07 bc	1.32 ± 0.01 ab
70 °C/4 min	10.82 ± 0.78 a	0.45 ± 0.01 bc	1.46 ± 0.08 a
80 °C/4 min	10.77 ± 0.48 a	0.48 ± 0.01 ab	1.33 ± 0.09 ab
90 °C/4 min	10.86 ± 1.35 a	0.48 ± 0.01 a	1.15 ± 0.08 cd
Two-way ANOVA (F-value) for treated groups only			
Conditioning temperature (3)	1.27	30.07 ***	13.84 ***
Retention time (1)	0.27	0.01	16.94 ***
Conditioning temperature × retention time (3)	0.96	7.18 **	4.60 *

RC, relativecrystallinity; *R*_1022/995_, ratio of peak intensity at 1022 and 995 cm^−1^; *R*_1047/1022_, ratio of peak intensity at 1047 and 1022 cm^−1^; ANOVA, analysis of variance. Data are presented as mean ± SD (*n* = 3). Different lowercase letters within a column indicate significant differences among all groups, including the untreated control, determined by Duncan’s multiple range test (*p* < 0.05). Two-way ANOVA (F-value) was performed on the steam-conditioned groups only to evaluate the main effects of conditioning temperature (df = 3), retention time (df = 1), and their interaction (df = 3). Significance levels: *** *p* < 0.001, ** *p* < 0.01, * *p* < 0.05.

**Table 3:** Effects of steam conditioning temperature and retention time on the pasting properties of swine compound feed.

Samples	Pasting Temperature (°C)	Peak Viscosity (cP)	Trough Viscosity (cP)	Breakdown (cP)	Final Viscosity (cP)	Setback (cP)
Control	75.78 ± 0.05 b	303.60 ± 10.26 d	217.33 ± 2.51 c	86.33 ± 7.77 c	251.00 ± 8.19 c	33.67 ± 5.86 c
60 °C/2 min	75.88 ± 0.18 b	284.67 ± 1.53 d	219.67 ± 1.52 c	61.67 ± 5.77 c	255.33 ± 4.73 c	32.33 ± 10.69 c
70 °C/2 min	75.83 ± 0.07 b	310.00 ± 5.00 d	238.00 ± 2.64 c	72.00 ± 3.46 c	295.67 ± 3.22 c	57.67 ± 1.16 c
80 °C/2 min	79.26 ± 0.49 a	638.00 ± 27.73 c	439.33 ± 33.38 b	198.67 ± 34.53 b	753.00 ± 134.58 b	313.67 ± 103.36 b
90 °C/2 min	79.13 ± 0.04 a	698.60 ± 60.21 c	448.67 ± 21.36 b	250.00 ± 38.97 b	781.00 ± 51.45 b	332.33 ± 30.35 b
60 °C/4 min	75.82 ± 0.02 b	296.00 ± 6.56 d	222.67 ± 0.57 c	73.33 ± 6.50 c	269.00 ± 2.65 c	46.33 ± 2.08 c
70 °C/4 min	75.83 ± 0.07 b	317.33 ± 5.13 d	224.33 ± 2.51 c	93.00 ± 2.65 c	260.67 ± 5.51 c	36.33 ± 4.93 c
80 °C/4 min	79.01 ± 0.02 a	785.33 ± 8.08 b	443.67 ± 8.50 b	341.67 ± 16.56 a	799.67 ± 45.61 b	356.00 ± 37.16 b
90 °C/4 min	79.07 ± 0.02 a	862.67 ± 13.05 a	525.33 ± 22.50 a	337.33 ± 10.12 a	1016.33 ± 76.53 a	491.00 ± 55.68 a
Two-way ANOVA (F-value) for treated groups only						
Conditioning temperature (3)	463.57 ***	676.80 ***	426.86 ***	217.19 ***	183.268 ***	110.36 ***
Retention time (1)	1.42	69.12 ***	6.85 *	65.20 ***	7.09 *	6.93 *
Conditioning temperature × retention time (3)	0.79	18.25 ***	8.97 **	14.28 ***	5.839 **	4.50 *

ANOVA, analysis of variance. Data are presented as mean ± SD (*n* = 3). Different lowercase letters within a column indicate significant differences among all groups, including the untreated control, determined by Duncan’s multiple range test (*p* < 0.05). Two-way ANOVA (*F*-value) was performed on the steam-conditioned groups only to evaluate the main effects of conditioning temperature (*df* = 3), retention time (*df* = 1), and their interaction (*df* = 3). Significance levels: *** *p* < 0.001, ** *p* < 0.01, * *p* < 0.05.

**Table 4:** Effects of steam conditioning parameters on the in vitro starch digestion profiles and hydrolysis kinetics.

Samples	RDS (%)	SDS (%)	RS (%)	Hydrolysis Parameter		
				*C*_∞_ (%)	*k ×* 10^2^ (min^−1^)	*R* ^2^
Control	32.31 ± 2.07 ab	23.70 ± 0.79 b	44.00 ± 2.27 a	68.61 ± 2.74 d	3.47 ± 0.32 bc	0.93
60 °C/2 min	33.12 ± 3.13 ab	37.64 ± 3.37 a	29.24 ± 3.93 cd	87.44 ± 3.27 ab	2.76 ± 0.35 c	0.96
70 °C/2 min	34.78 ± 4.36 ab	40.63 ± 4.86 a	24.59 ± 1.48 d	89.88 ± 4.74 a	3.19 ± 0.70 bc	0.98
80 °C/2 min	37.54 ± 0.46 ab	24.11 ± 6.39 b	38.36 ± 6.40 abc	81.53 ± 3.08 bc	3.03 ± 0.06 bc	0.93
90 °C/2 min	36.01 ± 4.55 ab	23.23 ± 3.44 b	40.76 ± 5.62 ab	79.08 ± 4.02 c	3.53 ± 1.05 abc	0.90
60 °C/4 min	29.44 ± 7.30 b	40.19 ± 12.41 a	30.37 ± 6.61 bcd	89.47 ± 3.30 a	2.69 ± 0.13 c	0.96
70 °C/4 min	35.63 ± 8.52 ab	35.91 ± 5.81 a	28.46 ± 9.35 cd	86.32 ± 6.20 abc	3.00 ± 0.45 bc	0.96
80 °C/4 min	41.05 ± 4.96 a	30.16 ± 2.99 ab	28.79 ± 7.60 cd	86.29 ± 5.37 abc	4.20 ± 1.56 ab	0.95
90 °C/4 min	39.96 ± 1.24 a	22.54 ± 3.00 b	37.50 ± 3.47 abc	82.99 ± 1.37 abc	4.62 ± 0.26 a	0.96
Two-way ANOVA (F-value) for treated groups only						
Conditioning temperature (3)	2.99	10.57 ***	4.83 *	4.37 *	3.80 *	-
Retention time (1)	0.32	0.10	0.63	1.10	2.69	-
Conditioning temperature × retention time (3)	0.73	0.86	1.41	1.21	1.45	-

RDS, rapidly digestible starch; SDS, slowly digestible starch; RS, resistant starch; *C*_∞_, maximum extent of hydrolysis; *k*, hydrolysis rate constant; *R*^2^, coefficient of determination; ANOVA, analysis of variance. Data are presented as mean ± SD (*n* = 3). Different lowercase letters within a column indicate significant differences among all groups, including the untreated control, determined by Duncan’s multiple range test (*p* < 0.05). Two-way ANOVA (*F*-value) was performed on the steam-conditioned groups only to evaluate the main effects of conditioning temperature (*df* = 3), retention time (*df* = 1), and their interaction (*df* = 3). Significance levels: *** *p* < 0.001, * *p* < 0.05.

## Data Availability

Data are contained within the article.

## Abbreviations

The following abbreviations are used in this manuscript:

ANOVA	Analysis of variance
CLSM	Confocal laser scanning microscopy
***C*****_∞_**	Ultimate starch digestibility
DG	Degree of gelatinization
DM	Dry matter
DSC	Differential scanning calorimetry
Δ*H*	Enthalpy of gelatinization
FTIR	Fourier transform infrared spectroscopy
k	Kinetic rate constant
RC	Relative crystallinity
RDS	Rapidly digestible starch
RS	Resistant starch
*T*_0_	Onset temperature
*T*_p_	Peak temperature
*T*_C_	Conclusion temperature
V-type	V-type crystalline pattern
XRD	X-ray diffraction
